# Prostate cancer diagnostics: evolution over 30 years and the impact of education level – a prospective population-based study

**DOI:** 10.3389/fonc.2025.1636292

**Published:** 2025-10-03

**Authors:** Leon Will, Erik Thimansson, Johan Bengtsson, Anders Bjartell, Sophia Zackrisson, Erik Baubeta

**Affiliations:** ^1^ Department of Imaging and Functional Medicine, Skåne University Hospital, Lund/Malmö, Sweden; ^2^ Diagnostic Radiology, Department of Translational Medicine, Lund University, Malmö, Sweden; ^3^ Department of Radiology, Helsingborg Hospital, Helsingborg, Sweden; ^4^ Diagnostic Radiology, Department of Clinical Sciences, Lund University, Lund, Sweden; ^5^ Division of Urological Cancers, Department of Translational Medicine, Lund University, Malmö, Sweden; ^6^ Department of Urology, Skåne University Hospital, Malmö, Sweden

**Keywords:** prostate cancer screening, diagnostic radiology, epidemiology, prostate cancer, mortality

## Abstract

**Objective:**

The aim of this study is to describe how the use of diagnostic imaging for prostate cancer (PCa) has evolved over time and to determine whether there are any differences in access to diagnostic imaging, type of cancers detected, and mortality based on the education level of patients.

**Methods:**

11,063 men were recruited between 1991 and 1996 and then prospectively followed until 2020. All new cases of PCa were recorded. At baseline, data on education level, heredity for cancer, and health status were collected. Incident PCa diagnoses during the study period were ascertained through record matching with national healthcare registers. The registers provided more detailed data on the cancer type and imaging performed.

**Results:**

1,816 men with diagnosed were PCa during the study period were included. No differences were seen between education levels in regard to access to diagnostic methods or tumour aggressiveness at diagnosis. Furthermore, no differences were seen in PCa-specific mortality, but there was higher overall mortality among individuals with a lower education level. During the study period, the use of plain radiographic examinations decreased, while the use of computed tomography (CT), prostate magnetic resonance imaging (MRI), and positron emission tomography/computed tomography (PET/CT) increased.

**Conclusion:**

Early detection and diagnostic methods for PCa have evolved over the last 30 years. In a healthcare system where men diagnosed with PCa had equal access to diagnostic pathways, no differences are seen in PCa specific mortality. Nevertheless, men with lower education level still had higher overall mortality.

## Introduction

Prostate cancer (PCa) is one of the most common cancers in the world, but while its incidence is increasing, mortality has decreased over the last 30 years (1, 2). Prior to the introduction of prostate-specific antigen (PSA) testing in the 1980s, patients were generally diagnosed after presenting with clinical symptoms or due to incidental findings at transurethral resection of the prostate (TURP). As PSA testing was performed more regularly and more asymptomatic cases of PCa were detected, an increase in incidence was seen (3). In parallel, improved imaging techniques using computed tomography (CT), bone scintigraphy, magnetic resonance imaging (MRI), and positron emission tomography/computed tomography (PET/CT) with increasingly advanced tracers have been introduced (4–6).

Historically, elevated PSA led to a physical examination, and in cases of remaining suspicion, core biopsies were performed (3). MRI was not routinely included in the diagnostic workup but was occasionally performed after repeated negative standard biopsies, for local staging, or as part of active surveillance. In recent years however, there has been a shift towards an "MRI first" approach, meaning that MRI examinations are conducted before patients undergo biopsies. This shift has led to an increase in targeted image-guided biopsies rather than standard systematic transrectal biopsies. This development arose in accordance with the conclusions of studies such as the PRECISION study, which demonstrated the benefits of MRI-targeted biopsies (7, 8).

Currently, there are no countries with ongoing population-based screening programs for PCa. Instead, a large proportion of PSA testing has historically been opportunistic, i.e. done on the initiative of the individual patient or physician (9). Socioeconomic factors are known to have an impact on participation in PSA testing, which could impact the outcome of the disease (10). High socioeconomic status correlates with better cancer outcomes, and for patients with PCa, this affects the chance of cancer detection and the time until curative treatment is received (11). Education level is also an important factor that is associated with socioeconomic status as it is determined early in life and deeply influences future employment, income, and usage of the healthcare system (12). Furthermore, education level is relatively easy to measure and tends to be stable over time.

Several studies have examined the advantages and disadvantages of national population-based screening programs for PCa (13–15). In 2018, the Swedish National Board of Health and Welfare decided not to implement nationwide screening but instead recommended organized testing, which in 2020 prompted several Swedish healthcare regions to initiate a digitally based organized PCa testing program (OPT). In this program, men between the ages of 50 and 74 years are offered a PSA test at prespecified intervals, and individuals with elevated PSA levels are then further examined with MRI (16, 17). As PSA-testing is offered free of charge to all male individuals in the eligible age group, the OPT project could in theory lead to more equal diagnostic and testing pathways. This is a well-founded assumption, but there is very limited research on how factors such as education level influence access to diagnostic examinations and whether it is relevant for PCa outcomes. In 2013, a Swedish group studied whether inappropriate use of diagnostic examinations had decreased over time and confirmed that such a trend was present (18). However, the study could not analyze which modalities were used or where the changes were found. The OPT project has since expanded and now covers almost the entire country.

In summary, little is known about how the use of diagnostic modalities has changed over time, or how education level influences access and outcomes. The aim of this study is therefore to describe the evolution of PCa diagnostics over 30 years in a prospective, population-based cohort, and to assess potential differences by education level as a proxy for socioeconomical status in order to later analyze the impact of the OPT project.

## Methods

**Figure 1:**
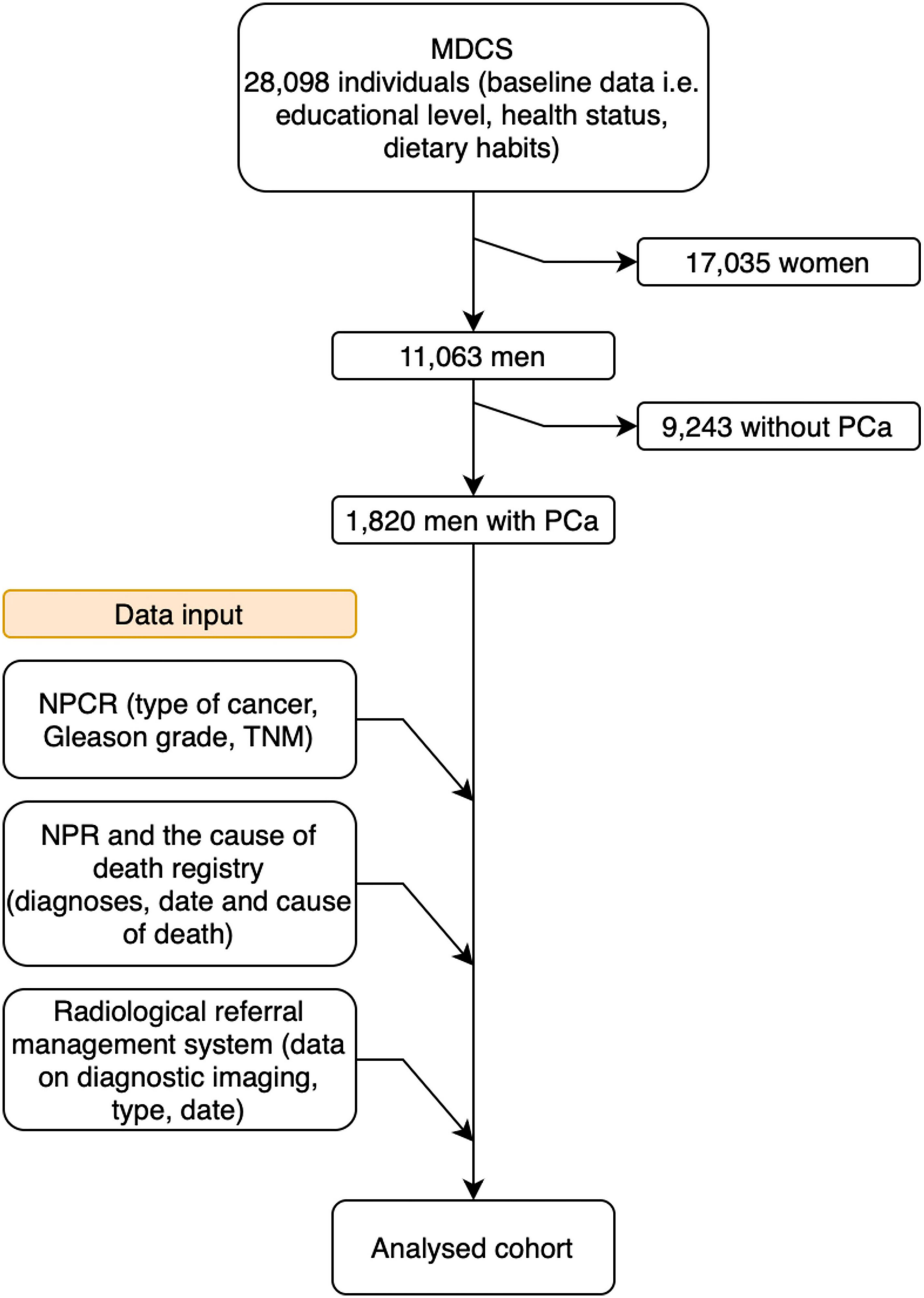
Flowchart of the data collection process. MDCS, Malmö Diet and Cancer Study; PCa. Prostate cancer; NPCR, National Prostate Cancer Register; TNM, Tumor, node, metastasis; NPR, National Patient Register.

This prospective population-based study included 11,063 men who were recruited between 1991 and 1996. No new individuals were added after 1996. Thus, the entire cohort was closed and followed until 2020. Data were collected from various diagnose specific and high-coverage healthcare registers. A flowchart of the data-collection process is presented in **Figure 1**.

### Cohort

Between 1991 and 1996, 28,098 individuals from the general population were recruited to the Malmö Diet and Cancer Study (MDCS) cohort. The aim was to study the relationship between diet and subsequent cancer risk. Participants answered extensive questionnaires about their educational background, health status, and dietary habits. They also underwent comprehensive health examinations. Details regarding the recruitment process of the cohort have been described previously (19).

The MDCS cohort included a total of 11,063 men. These men have been prospectively followed up, and data on any new diagnoses or deaths have been continuously collected from national mandatory health registers (mainly the National Patient Register (NPR) and the Swedish Cancer Register). The NPR has been shown to be highly valid, and it is mandatory for all healthcare providers to report to the registers (20). Within the NPR, all hospital-discharge diagnoses are documented according to International Classification of Diseases (ICD-9 and ICD-10) (21). Data on cancer diagnosis was retrieved from the Swedish cancer register, which is also a mandatory healthcare register with high validity and coverage of around 99% with documentation of all cancer diagnoses (22, 23).

Specific data concerning each patient's prostate cancer and how the patient entered the healthcare pathway, were collected from the Swedish NPCR: Gleason score and the tumor characteristics, locoregional lymph-node, and distant metastases (TNM) stage. The register was founded in 1998 and has been utilized increasingly. Since its creation, 235,000 PCa cases have been documented in the register (21, 24). The NPCR has been found to have high validity for PCa diagnoses and Gleason scores (25). The Gleason score was not available for patients diagnosed prior to 1997.

### Imaging modalities and ethical considerations

Data regarding imaging used were retrieved from the radiological referral management system. Data on the type of modality and number of examinations the individuals had undergone 6 months before and after the diagnosis of PCa were retrieved. All participants in the MDCS provided informed consent upon inclusion in the primary cohort, and ethical approval was given by the Ethics Committee of Lund University (LU-51-90, approved 1990). Ethical approval for the present study was obtained from the Swedish National Ethical Review Authority (approval number: 2021-05037, approved 2022).

### Statistical analysis

Descriptive statistics were used for the type of diagnostic imaging examination and stratified by education level. The cohort is historical, and the educational system changed between the time of the participants' education and the time of recruitment, so we sought to adjust their educational backgrounds to align with the modern system and make the education levels understandable in the current context. Furthermore, this prevents any group from being disproportionately different in size compared to the others. Therefore, education levels were divided into four categories based on the total years of education completed: primary education (up to 8 years), lower secondary education (9–10 years), upper secondary education (11–12 years), and at least one year beyond secondary education.

Cox regression analyses were used to study relative risks for overall mortality and cause-specific mortality (PCa). The models were adjusted for the year and age at diagnosis and country of birth. The cause-specific mortality analysis was verified by performing a competing-risk regression (Fine–Gray). Mortality data were also analyzed using Kaplan–Meier curves. All analyses were performed using R version 4.3.3 except for the competing-risk regression, which was performed using STATA SE version 18.

## Results

### Cohort

During the study period (1991–2020), 1,820 (16.7%) of the 11,063 men in the MDCS cohort were diagnosed with PCa. Full data on educational level was missing for 4 patients (0.2%) and the studied cohort therefore consisted of 1816 patients. The median follow-up time was 24.9 years, and the median age at diagnosis was 72.3 years (range 50–96 years). The largest study group (43.2%) had a primary level of education, while 18.4% had lower secondary education, 13.2% had upper secondary education and had graduated from high school, and 25.2% of the patients with PCa had some form of post-secondary education. The mean body mass index (BMI) was 26.1, and a majority (76.5%) either did not smoke or had quit smoking (**Table 1**).

**Table 1:** Demographics.

Variables	Patients (N = 1816)
Education (%)	
Primary education (≤8 years)	784 (43.2)
Lower secondary education (9–10 years)	335 (18.4)
Upper secondary education (11–12 years)	240 (13.2)
At least one year post-secondary education	457 (25.1)
Smoking (%)	
Yes, I smoke regularly	346 (19.0)
Yes, I smoke occasionally	81 (4.5)
No, I have stopped smoking	805 (44.2)
No, I have never smoked	588 (32.3)
Heredity for cancer	
Father with cancer	442 (24.3)
Mother with cancer	399 (21.9)
Sibling with cancer	256 (14.1)
Born in Sweden	1629 (89.5)
		Age at inclusion [mean (SD)]	59.53 (6.93)
Height at inclusion [mean (SD)]	176.86 (6.54)
Weight at inclusion [mean (SD)]	81.63 (11.46)
BMI at inclusion [mean (SD)]	26.08 (3.25)
Age at diagnosis [mean (SD)]	72.61 (7.26)
Time to diagnosis (years) [median (range)]	12.32 [0.06, 29.62]
Follow-up time (years) [median (range)]	24.87 [1.22, 29.79]

### Imaging

**Figure 2:**
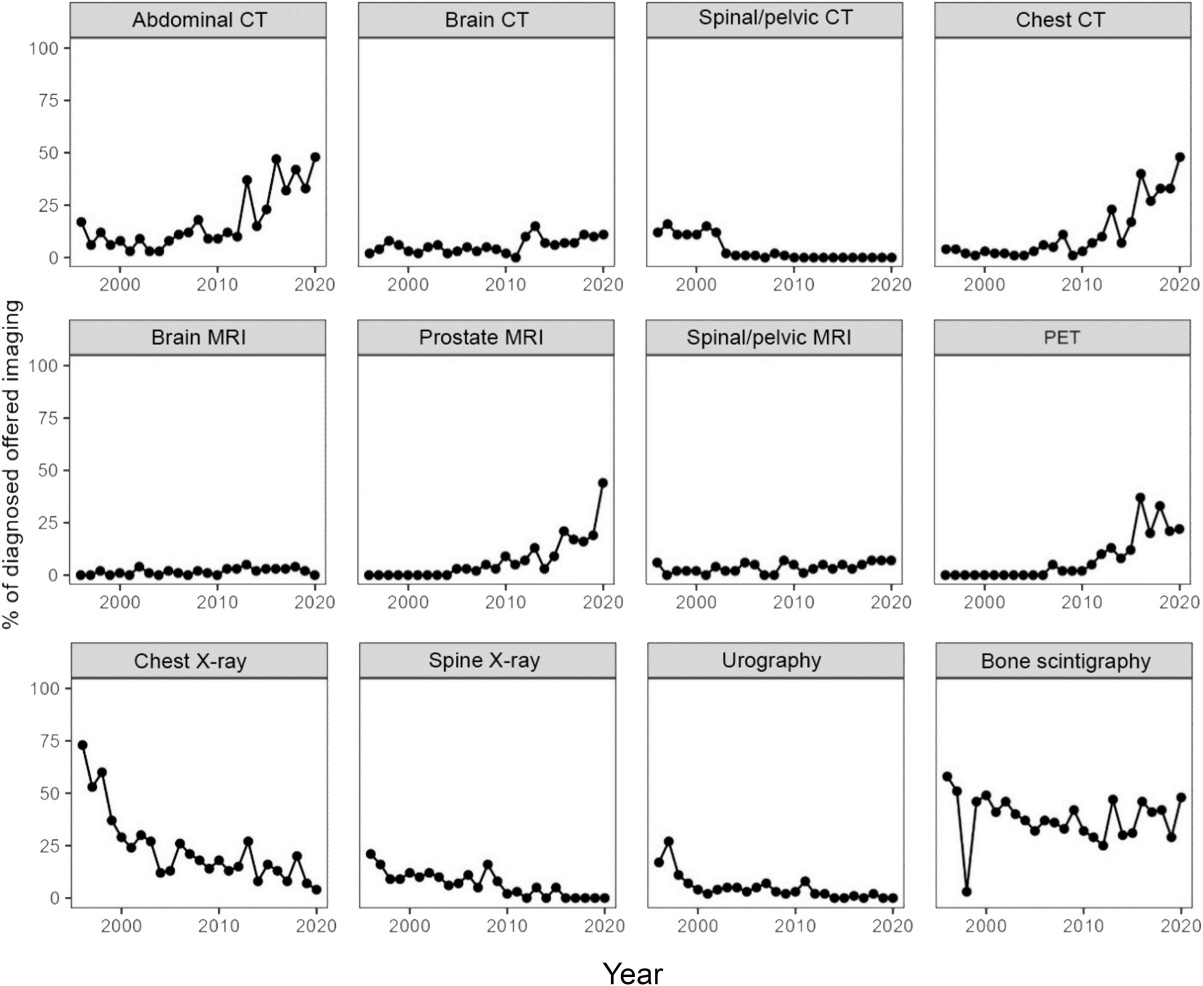
The proportion of diagnosed patients per year who received each type of imaging in conjunction with their diagnosis.

In total, the patients with PCa underwent 73,343 diagnostic imaging examinations, of which 2,279 were associated with their primary cancer diagnosis. Throughout the follow-up period, the use of plain radiographic examinations declined, whereas the use of CT, prostate MRI, and PET/CT increased. The proportion of bone scintigraphy remained constant during the study period (**Figure 2**). The use of PET/CT increased later in the study period, and in total, 115 PET/CT examinations were performed. A smaller proportion of these were prostate-specific membrane antigen [¹^8^F]PSMA-1007 PET/CT (10 examinations (8.7%), all performed in 2019 and 2020), but the clear majority were [¹¹C]choline PET/CT. For a minority of the cases before 2019, no exact information on the used tracer is available and it cannot be excluded that other radiopharmaceuticals such as [¹^8^F]fluorocholine, [¹^8^F]NaF, [¹^8^F]FDG) were used.

### Impacts of education level

**Figure 3:**
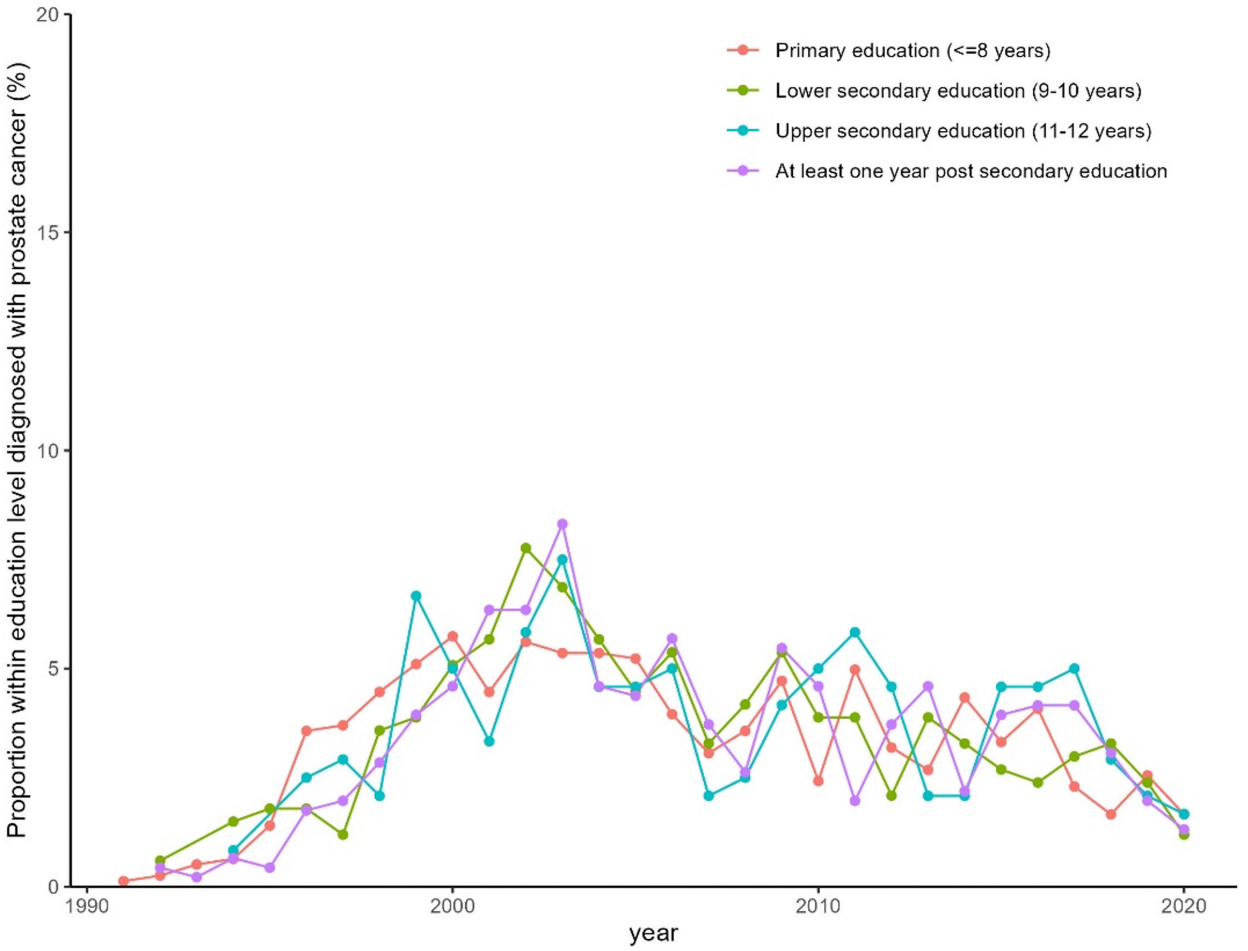
Proportion of men in the MCDC diagnosed with prostate cancer per year stratified by education level.

**Figure 4:**
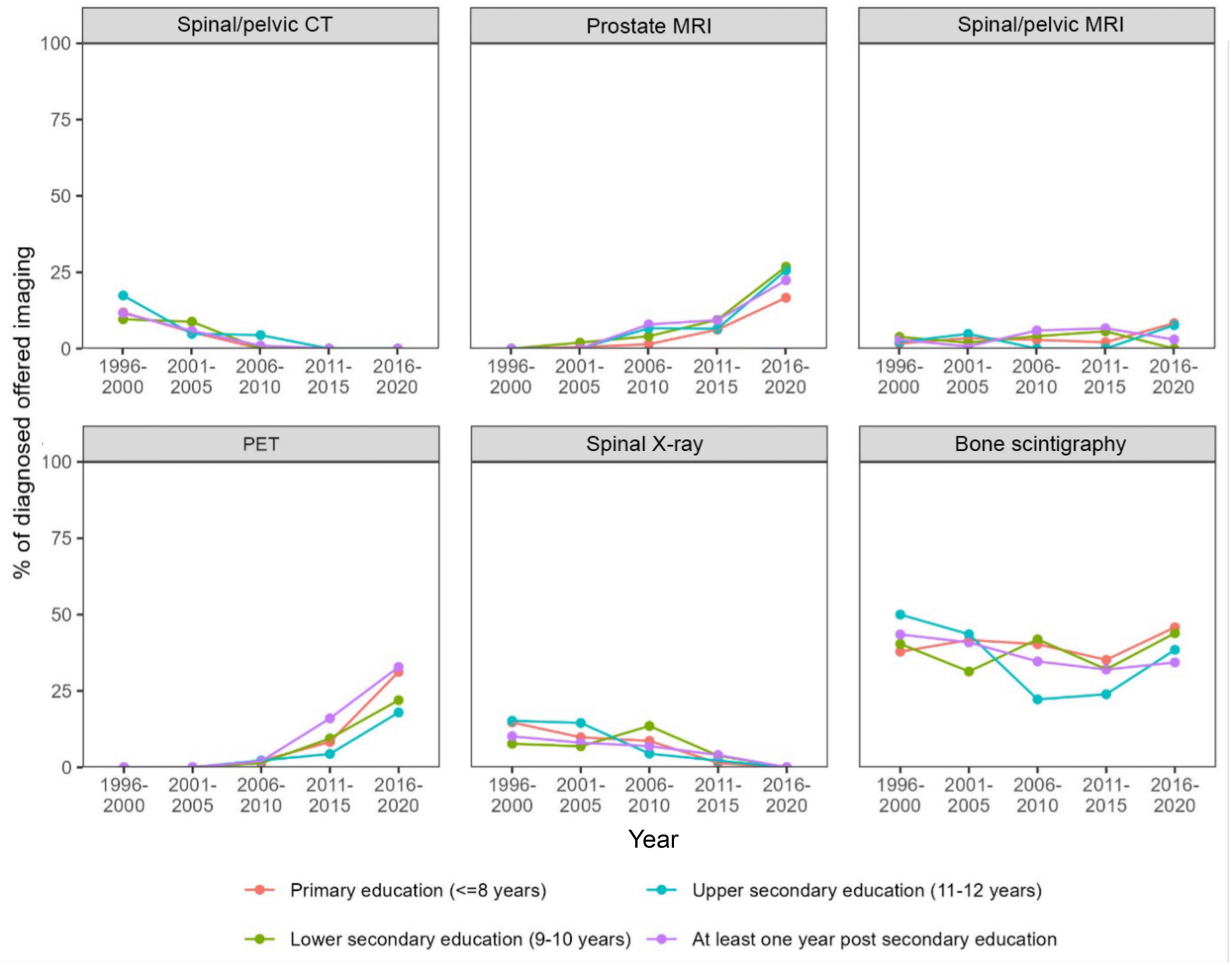
Proportion of diagnosed patients per year who received each type of imaging diagnostic at the time of diagnosis stratified by education level.

The proportion of men diagnosed with a PCa during the study period was equally distributed between different education levels. More patients with higher education entered the healthcare pathway through a healthcare examination (48.9% in the group with highest education vs. 40.6% in the group with the lowest education level). Correspondingly, the group with the highest education entered less frequently with lower urinary tract symptoms (19.6% for the highest education group vs. 27.3% for the group with lowest education) (**Table 2**). Furthermore, there were no differences in the type of diagnostic examinations used for the PCa diagnosis (**Figures 3**, **4**). No differences were seen in the severity of the Gleason score between the education groups. A majority were found to have a Gleason score of 7 or higher. No Gleason score was available for 355 patients (19.5%) (**Table 3**).

**Table 2:** The number (and proportion) of men within each educational level and the corresponding seeking pathway which led to the prostate cancer diagnosis.

Education level	Seeking pathway			Missing %
	Health examination	LUTS	Other clinical symptoms	
Primary education (<=8 years)	222 (40.4%)	150 (27.3%)	178 (32.4%)	29.8
Lower secondary education (9–10 years)	104 (40.6%)	74 (28.9%)	78 (30.5%)	23.6
Upper secondary education (11–12 years)	65 (37.6%)	49 (28.3%)	59 (34.1%)	27.9
At least one year post-secondary education	172 (48.0%)	71 (19.8%)	115 (32.1%)	21.7

LUTS, lower urinary tract symptoms.

**Table 3:** The number (and proportion) of men within each education level with the corresponding Gleason score.

Education level	Gleason score				Missing %
	<7	7	8	9–10	
Primary education (≤8 years)	256 (42.0%)	195 (32.0%)	56 (9.2%)	103 (16.9%)	22.2
Lower secondary education (9–10 years)	115 (42.1%)	82 (30.0%)	31 (11.4%)	45 (16.5%)	18.5
Upper secondary education (11–12 years)	79 (40.7%)	60 (30.9%)	25 (12.9%)	30 (15.5%)	19.2
At least one year post-secondary education	173 (45.1%)	125 (32.6%)	29 (7.6%)	57 (14.8%)	16.0

Total n = 1816. Data missing for 355 (19.5%).

**Figure 5:**
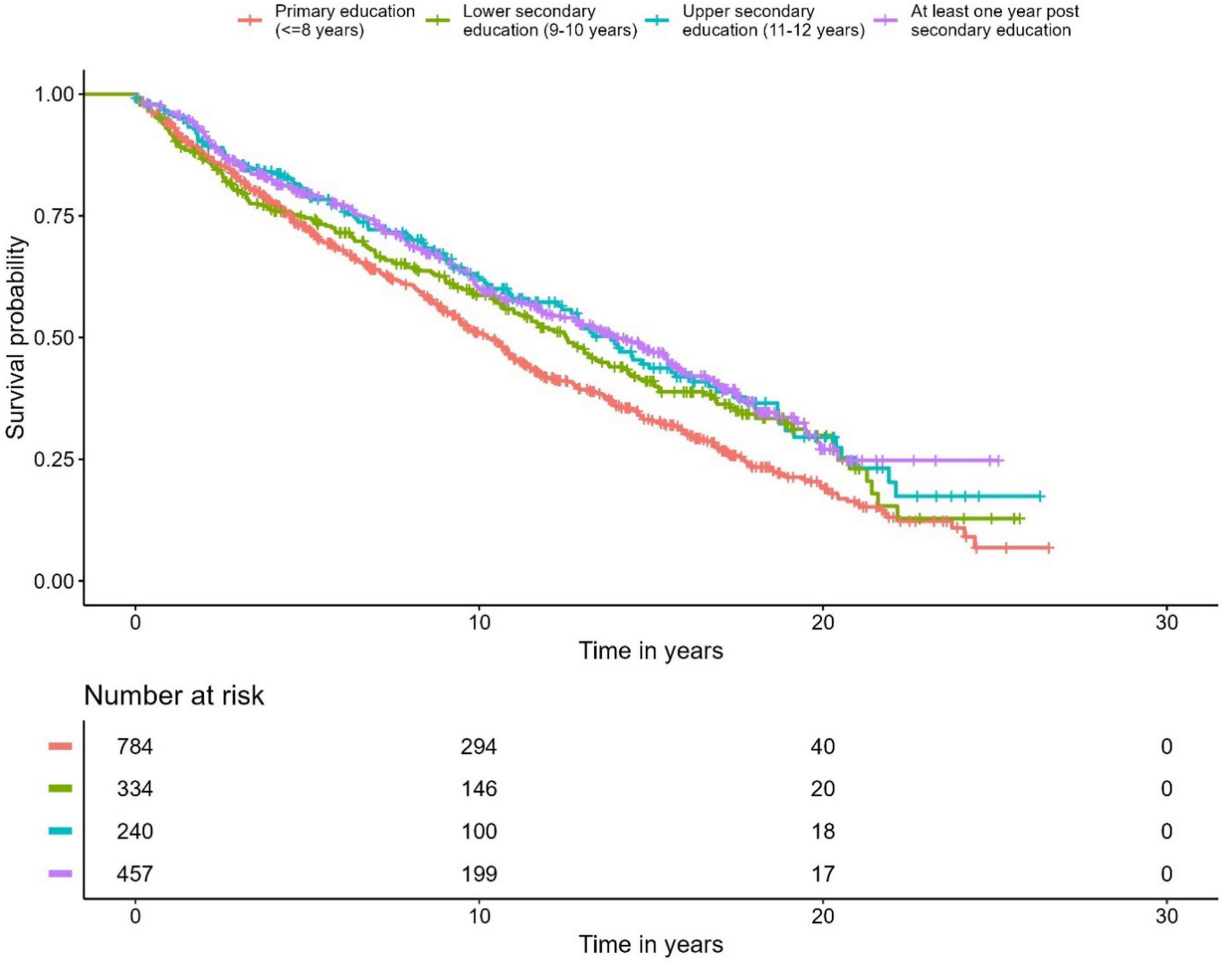
Kaplan-Meier curve of overall survival, stratified by educational level.

**Figure 6:**
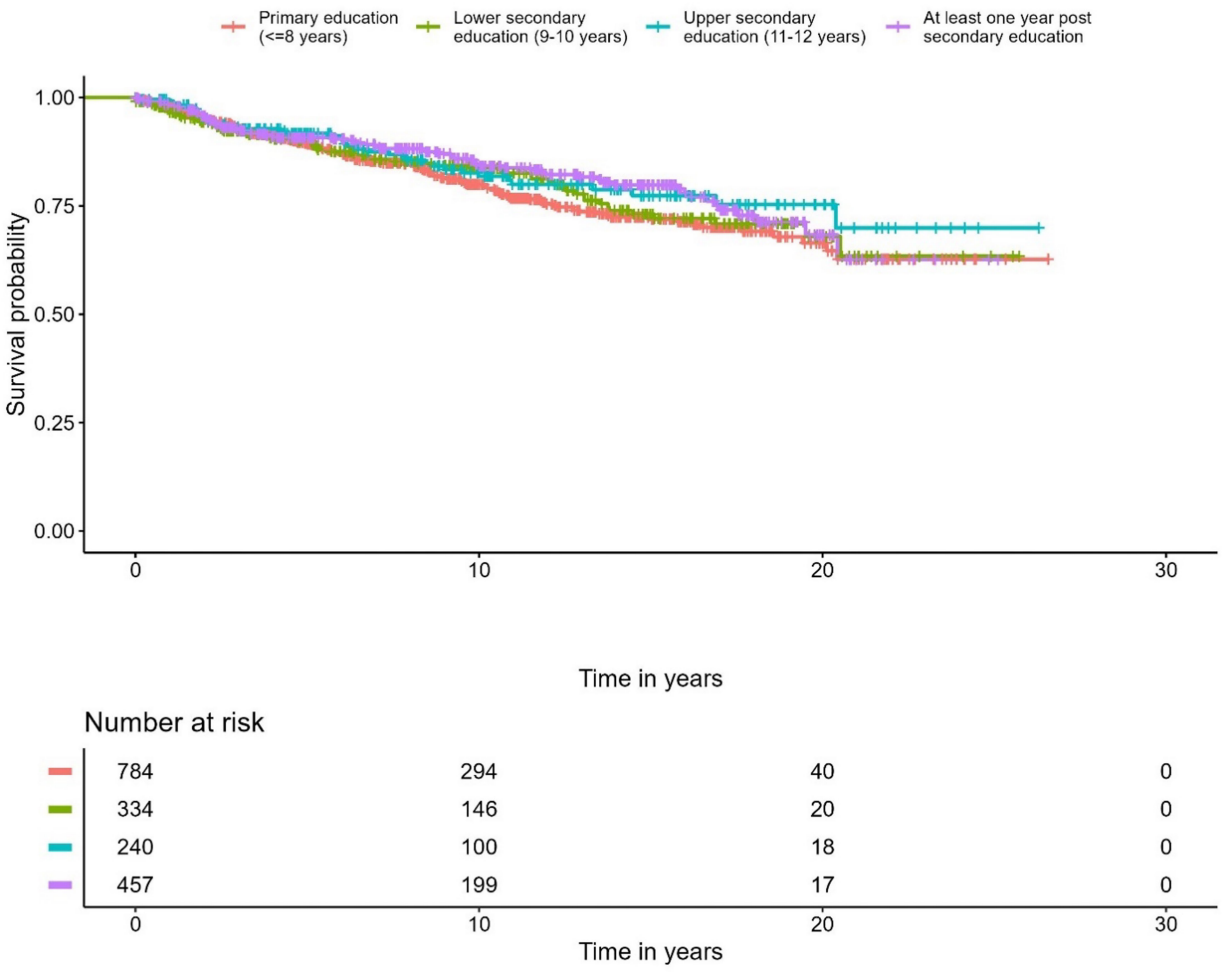
Kaplan-Meier curve of cause specific mortality for prostate cancer, stratified by educational level.

A correlation was observed between education level and overall survival. In the Cox regression model, men with the highest education level had 28% lower overall mortality compared to the group with the lowest education level (*p* < 0.001) (**Figure 5**, **Table 4**). No differences were seen in the PCa-specific survival analysis between education levels (**Figure 6**, **Table 5**). Further, a competing risk regression was performed to rule out impacts of competing risks on the result, but the analysis did not show any significant differences.

**Table 4:** Hazard ratios for unadjusted and adjusted Cox regression (unadjusted and adjusted for age at diagnosis, year of diagnosis and country of birth).

Education level	Unadjusted model		Adjusted model	
	HR (95% CI)	P-value	HR (95% CI)	P-value
Primary education (≤8 years) (ref)	1	–	1	–
Lower secondary education (9–10 years)	0.82 (0.69; 0.97)	0.019	0.89 (0.75; 1.05)	0.167
Upper secondary education (11–12 years)	0.72 (0.59; 0.88)	0.001	0.75 (0.61; 0.91)	0.004
At least one year post-secondary education	0.71 (0.61; 0.83)	<0.001	0.77 (0.65; 0.90)	0.001

**Table 5:** Hazard ratios for unadjusted and adjusted Cox regression for cause specific mortality (unadjusted and adjusted for age at diagnosis, year of diagnosis and country of birth).

Education level	Unadjusted model		Adjusted model	
	HR (95% CI)	P-value	HR (95% CI)	P-value
Primary education (≤8 years) (ref)	1	–	1	–
Lower secondary education (9–10 years)	0.92 (0.68; 1.24)	0.578	1.01 (0.75; 1.36)	0.934
Upper secondary education (11–12 years)	0.79 (0.55; 1.12)	0.185	0.84 (0.59; 1.19)	0.328
At least one year post-secondary education	0.79 (0.60; 1.04)	0.099	0.88 (0.67; 1.17)	0.390

## Discussion

There are three main findings in this study. First, no differences were seen between education levels in regard to access to diagnostic methods, tumor aggressiveness, or PCa-specific mortality. Second, despite the first finding, there was still higher overall mortality among individuals with a lower level of education. Lastly, unsurprisingly, the use of plain radiographic examinations decreased, while the use of CT, MRI, and PET/CT increased between 1991 and 2020.

The Swedish healthcare system is almost exclusively publicly funded with patients paying a low fee, while the remainder is financed through taxes. But even in such a system, the patient fees and education level can influence whether individuals seek medical care and their access to it (26, 27). PCa among individuals in this study were not detected through organized testing but in clinical routine, including a variety of unorganized detection pathways, such as opportunistic PSA testing, clinical symptoms, or incidental examination findings (i.e. after TURP). We observed modest differences in healthcare seeking pathways across educational levels, with health examinations being a more common route of detection among patients with higher education. Our study did not identify any differences in access to diagnostic methods, severity of detected PCa or cause specific mortality. One of the primary goals of implementing the OPT in Sweden was to ensure more equal access to PCa diagnostics (16).

An early study that analyzed the influence of socioeconomic factors on participation in OPT showed that socioeconomic factors may have an impact (28). These findings are consistent with those of other studies analyzing the influence of socioeconomic factors on participation in other cancer screening programs (29). These results are not surprising as we know that socioeconomic disparities in accessing healthcare are determined not only by financial means to obtain care, but also by how individuals process information and the resources they possess to translate that knowledge into concrete actions. There are also studies showing the opposite, where little or no associations were seen between socioeconomics and access to PCa diagnostics or care (30). One interpretation of the results could be that there are greater barriers to overcome in encouraging men to seek healthcare rather than differences in the care they receive once they are within the healthcare system. Therefore, these somewhat contrasting results are interesting in the context of the new OPT system and how to evaluate its results.

In recent years, there has been a significant shift in the diagnostics of PCa towards an "MRI first" approach. This shift gained momentum in the years preceding 2020, partly due to the findings from the PRECISION and Gothenburg studies (7, 13, 14).

Additionally, the PI-RADS diagnostic algorithm, which uses MRI for assessment, has been modified and increasingly adopted in prostate diagnostics (31). Recent evidence further supports this diagnostic approach. PI-RADS v2 combined with PSA density has shown high predictive value for clinically significant prostate cancer in biopsy−naïve patients (32). A potential benefit of this shift is a reduction in the numbers of biopsies of indolent tumors as combining systematic with MRI−targeted biopsy achieves the highest detection rates overall (33). MRI remains reliable even in men with prior negative biopsies, delivering detection rates comparable to biopsy−naïve populations (34). While this "MRI first" approach was partially evident towards the end of our study period, it had not yet been fully implemented. Nevertheless, a tendency towards more MRI in the later study years can be observed in our results.

Another tendency that was noticeable in later years is the increasing use of [¹^8^F]PSMA-1007 PET/CT in in more advanced disease or cases of PSA persistence after prostatectomy, which may impact treatment and survival (35, 36). The [¹^8^F]PSMA-1007 PET/CT examinations performed in our cohort were all carried out in the two last years (2019 and 2020). The use of conventional radiographic methods also declined which reflects the developments in diagnostic practice.

Interestingly, while we noted a difference in overall survival based on education level, there was no difference in PCa-specific mortality. This indicates that equal access to healthcare can eliminate survival differences for specific disorders. At the same time, socioeconomic status is multifactorial, which is represented in the survival differences between the observed education-level groups. The higher all-cause mortality in lower education groups likely reflects differences in health behaviors (e.g. smoking, adiposity), comorbidity burden, and social determinants of health. This interpretation is consistent with prior Swedish and international findings (11, 37, 38). Although the results are specific to a sub-population in Sweden, they are of interest to many other healthcare systems as a large number of the world's systems are either publicly funded or have a broad public health-insurance network (39). Detailed exploration of these factors was beyond the scope of our study, which focused on diagnostic access, but we emphasize this gradient as an important public health concern.

### Study limitations and strengths

There are several limitations to this study. First, the cohort was closed. Although the included individuals had a spread in age, no new individuals were recruited after 1996. Consequently, the cohort as a whole aged over time, and since increased age is a risk factor for cancer development, there has been a gradual increase in risk within the cohort. On the other hand, the age distribution of the cohort has contributed to a relatively even spread of incident cancer cases over the follow-up period, which can be seen as a strength, given the study's aim to analyze the development of PCa over time. Second, Gleason scores were not recorded in the Swedish National Prostate Cancer Register prior to 1997, which limits the ability to assess tumor aggressiveness for the earliest cases. However, the majority of prostate cancer diagnoses in this cohort occurred after 1997, meaning Gleason scores were available for most patients. Third, the study cohort differed slightly from the general population of Malmö in that the education level was somewhat higher and the proportion of foreign-born men lower (19). These differences may bias our findings toward underestimating disparities, since both higher education and native birth are associated with greater healthcare access and improved outcomes and the results of the present study may therefore not fully reflect inequalities present in the broader population. However, this probably influences the outcome to only a limited extent since the results were internally compared within the cohort and stratified by education level.

Moreover, while we report differences in overall mortality by education level, our analyses were not designed to assess the contributions of lifestyle, comorbidity, or other social determinants. This question has been addressed in prior studies but remains relevant for interpreting our results.

For the performed PET/CT examinations, tracer metadata were incompletely recorded for early examinations. Where available, [¹¹C]choline predominated. [¹^8^F]PSMA-1007 PET/CT was introduced in 2019 and dominated from that point onward. The main strengths of this study lie in the prospective nature of the cohort. The study population was recruited as a cross-section of the population approximately 30 years ago. Information on lifestyle habits, which is often difficult to obtain retrospectively, was collected at the start of the study. Therefore, the data on these variables can be considered particularly reliable. Other important variables for the study, such as data on imaging diagnostic methods, were obtained from the healthcare digital medical-record system and can therefore be considered complete.

## Conclusion

Early detection and diagnostic methods for PCa have evolved over the last 30 years. In a healthcare system where men diagnosed with PCa had equal access to diagnostic pathways, no differences are seen in PCa specific mortality. Nevertheless, men with lower education level still had higher overall mortality.

## Data Availability

The cohort comes from a prospective study which followed individuals over 30 years. Since data contains personal identification it can't be made publicly available but will be available upon formal request with legal and ethical review. Requests to access these datasets should be directed to https://www.malmo-cohorts.lu.se.
